# Active Peripheral Osteoma in the Mandibular Notch: A Longitudinal Clinical Report

**DOI:** 10.1155/2022/6364599

**Published:** 2022-03-22

**Authors:** Jun Sasaki, Shogo Hasegawa, Hitoshi Miyachi, Toru Nagao

**Affiliations:** ^1^Department of Maxillofacial Surgery, School of Dentistry, Aichi-Gakuin University, 2-11 Suemori-Dori, Chikusa, Nagoya, Aichi 464-8651, Japan; ^2^Department of Maxillofacial Surgery, Okazaki City Hospital, 3-1 Goshoai, Koryujicho, Okazaki, Aichi 444-8553, Japan

## Abstract

Osteomas are benign osteogenic tumors composed of mature compact or cancellous bone and are characterized by slow, painless growth. A peripheral osteoma located in the mandibular notch is extremely rare. Here, we describe the case of a 38-year-old woman with an active peripheral osteoma in the mandibular notch for long-term follow up. Surgical resection of the lesion was performed, which resulted in the improvement of the mandibular function and temporomandibular dysfunction. This is the first report of resection surgery after confirming the activity of peripheral osteoma by bone scintigraphy. Creating a three-dimensional model of the mandible promises an accurate surgical plan.

## 1. Introduction

Osteomas are benign tumors composed of mature compact or cancellous bone and are characterized by slow, painless growth [1–3]. They can be peripheral, central, or extraskeletal [4, 5]. Most peripheral osteomas are known to occur in the mandibular body, angle, condyle, or coronoid process [2, 4, 5]. The occurrence of a peripheral osteoma in the mandibular notch is extremely rare, with an incidence of 1.6% among mandibular peripheral osteoma cases [4, 5]. Although several studies have described peripheral osteomas of the jawbone, only eight cases of peripheral osteomas in the Sigmoid notch of the mandible have been reported.

We report the case of an active peripheral osteoma in the mandibular notch of a 38-year-old woman in whom we were able to clarify the temporal course of the disease.

## 2. Case Presentation

**Figure 1:**
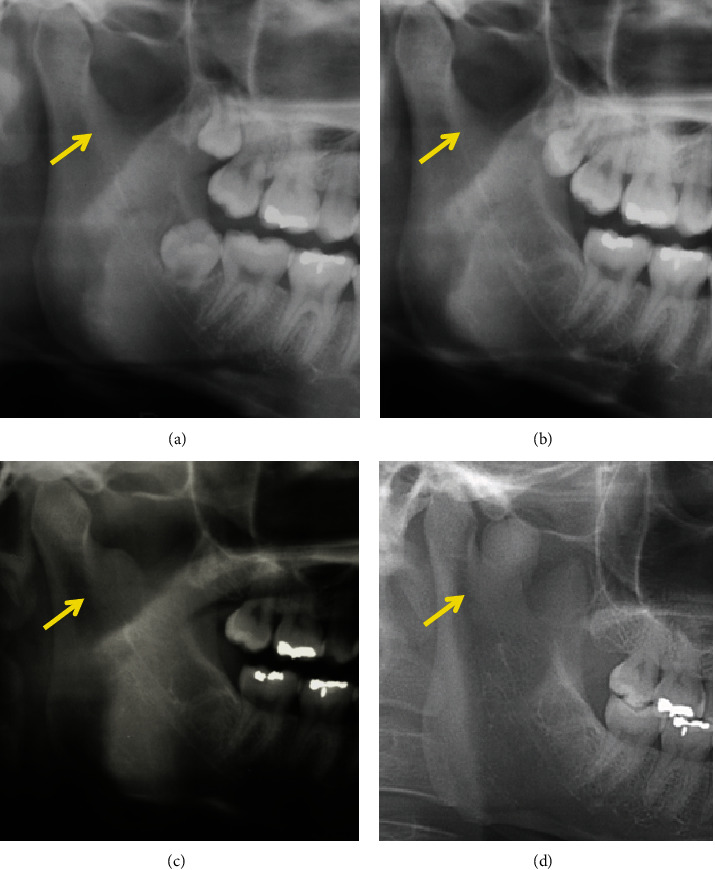
Time course of osteoma in the right mandibular notch by panoramic radiography. (a) At the age of 18, no osteoma was found in the right mandibular notch. (b) At the age of 21, no osteoma was found in the right mandibular notch. (c) At the age of 26, a bony bulge was found in the right mandibular notch. (d) At the age of 38, an oval radiodense mass was observed in the right mandibular notch.

**Figure 2:**
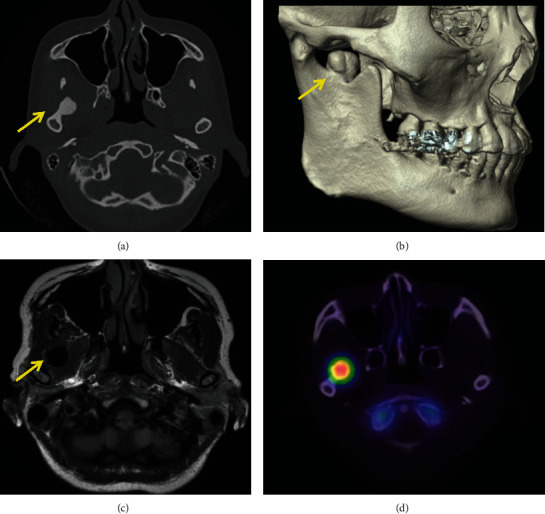
Preoperative examination. (a) Computed tomography showed a 13.3×12.8×10.0mm sized lesion showing Hounsfield units (HU) equivalent to that of the mandible, arising from the right mandibular notch. The CT values of osteoma ranged from 1480 to 1580 HU. (b) 3D-CT showed osteoma was found in the right mandibular notch. (c) Magnetic resonance imaging showed uniform hypointense trabecular bone, and there was no continuity of fat signals in the bone marrow, suggesting exclusion of the lateral pterygoid muscle because of bone lesions. (d) Radioisotope imaging showed significant uptake of Tc-99m by the osteoma in the right mandibular notch.

**Figure 3:**
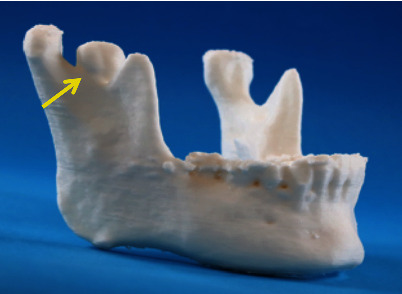
Three-dimensional model of the mandible. We created a three-dimensional model of the mandible to facilitate an explanation to the patient resection surgery using an intraoral approach was planned.

A 38-year-old woman with complaints of pain during mandibular movement was referred to our department by a dental clinic. She had no significant medical history, and she was not taking any medication. The patient was evaluated based on preoperative clinical manifestations, panoramic radiography, computed tomography (CT), magnetic resonance imaging (MRI), and radioisotope imaging (RI). Panoramic radiography revealed a circular radiopacity between the right mandibular condyle and the muscle process (Figure 1(d)). On reviewing the patient's panoramic radiograph taken at the age of 18 and 21 years, no osteoma was found in the mandibular notch (Figures 1(a) and 1(b)). At the age of 26, a bone bulge was found in the right mandibular notch (Figure 1(c)). CT showed a 13.3×12.8×10.0mm lesion showing Hounsfield units (HU) equivalent to that of the mandible, arising from the right mandibular notch (Figures 2(a) and 2(b)). MRI showed uniform hypointense trabecular bone, and there was no continuity of fat signals in the bone marrow, suggesting the exclusion of the lateral pterygoid muscle due to bone lesions (Figure 2(c)). RI showed significant uptake of Tc-99m (Figure 2(d)). Bone scintigraphy showed circular area of strong radioisotope accumulation with indistinct boundaries, consistent with lesion in right mandibular notch. No neurological symptoms of the mandible were observed, and there was no history of trauma or infection in the maxillofacial region. No abnormalities were found on blood or urine analysis. Based on these findings, the diagnosis of an active peripheral osteoma was suspected. Using the CT data and Mimics software^®^ (version 23.0, Materialise, Leuven, Belgium), we created a three-dimensional (3D) model of the mandible (Figure 3) to facilitate an explanation to the patient. Resective surgery using an intraoral approach was planned. A fused deposition modeling 3D printer (da Vinci Mini w Plus, XYZ printing, Taiwan) and a thermoplastic resin (polylactic acid filament) were used to prepare the model.

**Figure 4:**
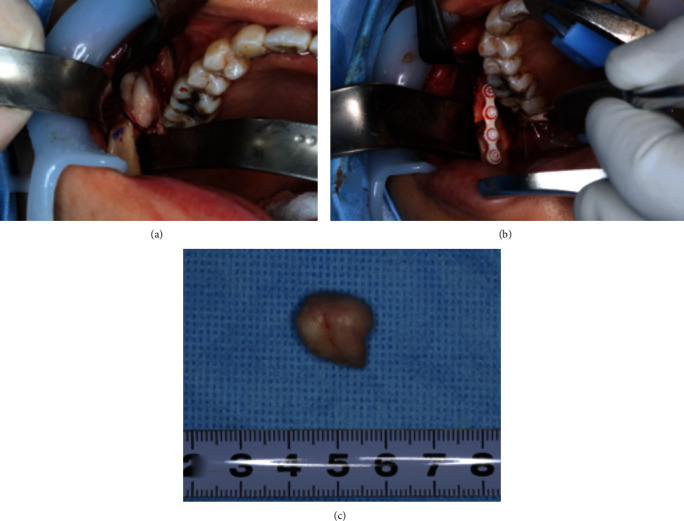
Intraoperative view. (a) The lesion was exposed by an intraoral approach. (b) The myoprojection was temporarily dissected, and the bone was repositioned and fixed with an absorbent plate. (c) Excised osteoma.

**Figure 5:**
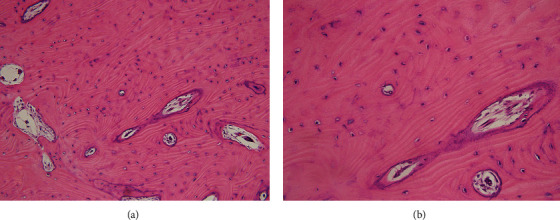
The histopathology of the peripheral osteoma. (a) Microscopic features of peripheral osteoma (hematoxylin-eosin, original magnification ×200). (b) Microscopic features of peripheral osteoma (hematoxylin-eosin, original magnification ×400).

On June 23, 2021, the entire tumor, including the portion that demonstrated Tc-99m uptake, was resected under general anesthesia. The myoprojection was temporarily dissected (Figure 4(a)), and the bone was repositioned and fixed with an absorbent plate (Super FIXSORB^®^) (Figure 4(b)). The excised mass measured 13×12.0×10.0mm with a weight of 2.7 g (Figure 4(c)). On histologic examination, osteomas are composed of dense, mature, predominantly lamellar bone. The osteoma was diagnosed an actively growing lesion (“hot”) with radionuclide bone imaging, interosseous spaces composed of fibrous, fibrovascular, and lined by osteoblasts within a well-vascularized moderately cellular fibrous stroma (Figure 5). The case presented here was diagnosed as a compact osteoma because of its characteristic microscopical features, radiographic findings, and clinical features. Immediately after the operation, there was a slight trismus with no other complications. There were few functional changes and minimal temporomandibular joint dysfunction. The patient was followed-up for 5 months, and no recurrence was noted.

## 3. Discussion

Peripheral osteomas, also known as periosteal osteomas, most frequently involve the craniofacial bones and are the most common benign tumors of the paranasal sinuses. Peripheral osteomas of the jawbones are however uncommon [2]. The origin of peripheral osteoma onset is unknown, and it has been suggested that it results from a reaction to trauma and infection [2]. However, there was no history of infection or trauma in the present case. The osteoma may have slowly developed during the age of 21–26 years after the cessation of mandibular growth and development of secondary sexual characteristics. Surgery is considered for large, deforming osteomas [1, 6]. In the present case, surgical resection was considered owing to the progressive nature of the lesion confirmed by RI.

Only eight cases of osteomas in the mandibular notch have been reported so far [1, 6–10], and the present case is the ninth (Table 1). In the previous cases, the patients' age ranged from 16 to 78 years, with a male-to-female ratio of 1 : 3.5. The location of the osteoma was medial in four cases, lateral in four cases, and medial and lateral in one case. The lesion developed in the right mandibular notch in five cases and in the left mandibular notch in four cases. The patients displayed symptoms of swelling in four cases and pain in two, with no symptoms in three cases. Of the five surgically treated cases of osteoma, four were intraoral approaches. This is the first case to use RI to confirm the dynamic state of the osteoma; RI is therefore useful for diagnosing osteomas.

In addition, a 3D model was created based on the preoperative CT data using a fused deposition modeling 3D printer and a thermoplastic resin (polylactic acid filament). The 3D model is useful for preoperative patient education and surgical planning. In this case, because a 3D model was created only for the mandible, the surgery of the maxillary tuberosity and maxillary teeth was restricted, and it was difficult to predict. Among the reports of osteomas in the mandibular notch, there were descriptions of intraoral surgery in four cases. In the present case, considering the patient's aesthetics, we approached the lesion intraorally.

Currently, the number of reported cases of peripheral osteoma in the mandibular notch is small, and many aspects of this entity remain unclear, including the treatment method and prognosis. Further accumulation and examination of cases are expected.

## 4. Conclusions

We encountered a case of an active peripheral osteoma in the mandibular notch, which has rarely been reported in the literature. A dynamic diagnosis of peripheral osteoma was made possible using composite imaging. It was easy to obtain anatomical orientation in the surgical field through preoperative surgical simulation using a 3D model. An osteoma in the mandibular notch can be resected by an intraoral approach, in which case it is desirable to reposition the myoprojection.

## Figures and Tables

**Table 1:** Reported cases of peripheral osteoma in the mandibular notch.

No.	Reference	Year of publication	Age (years)	Sex	Location	Side	Size (in mm)	Symptoms	Treatment
1	Present case	2021	38	Female	Medial	Right	15×14×10	Pain	Surgery (intraoral)
2	*Nakayama* et al. [3]	2021	69	Female	Lateral	Left	25×20×15	Swelling	Surgery (extraoral)
3	*Bocchialini* et al. [1]	2018	37	Female	Medial and lateral	Left	15×13×11	Pain	Surgery (intraoral)
4	*Sasidharan* et al. [6]	2015	52	Female	Lateral	Left	30×25×20	Swelling	Surgery (intraoral)
5	*Rao* et al. [7]	2013	16	Female	Lateral	Right	30×30	Swelling	Surgery (intraoral)
6	*Iwai* et al. [4]	2013	78	Female	Medial	Right	36×35×30	None	Follow-up
7	*Sekerci* et al. [8]	2011	59	Male	Medial	Right	Not available	None	Follow-up
8	*Schulze* [9]	2008	73	Female	Medial	Left	30×30×27	Pain	Unknown
9	*Bessho* et al. [10]	1987	26	Male	Lateral	Right	34×29×17	Swelling	Surgery
